# Symphysiotomy

**Published:** 1899-03-11

**Authors:** 


### SYMPHYSIOTOMY.

Dr. Ida Balfour describes[1] a case in which symphysiotomy for contracted pelvis was followed by nearly normal labour two years later. The patient was a Mohammedan woman between 30 and 40 years of age. At her first confinement, after much trouble, the civil surgeon had been called in, and had performed craniotomy. In the second confinement it was found on being called that the membranes had been ruptured four hours, the os was fully dilated, and the head presenting in the second position. The pelvis was contracted, and forceps failed to deliver. Symphysiotomy was therefore performed by the subcutaneous method. "Before the knife had completely cut through the cartilage the joint gave way with a crash. Forceps were reapplied, and a good-sized boy delivered. The opening in the skin was at once dressed and a firm bandage applied to the hips." The operation does not seem, however, to have been entirely without drawbacks, for it was followed by a sinus, which went on discharging for some time. She also had "white leg" on the left side, and pains in the right leg with considerable muscular atrophy. From these, however, she recovered, and two years later was easily delivered of a girl by aid of forceps. "The case is interesting, as showing a permanent improvement in the size of the pelvis after the operation of symphysiotomy. I was also struck," says the writer, "by the ease of the operation (only a knife and a catheter were used), making it eminently suitable for small, dark, and dirty houses, where no skilled assistance can be had." We think, however, that the latter deduction must be taken with considerable reservation.

[1] Brit. Med. Jour., Feb. 18.

